# Supplementary material for: Intense Pulsed Light Attenuates UV-Induced Hyperimmune Response and Pigmentation in Human Skin Cells
Source: Int J Mol Sci. 2021 Mar 20;22(6):3173. doi: 10.3390/ijms22063173 (PMC8003787; doi:10.3390/ijms22063173)
Supplement: Supplementary file 1 [file ijms-22-03173-s001.pdf]

Supporting Information

Intense pulsed light attenuates UV induced hyper-immune and pigmentation in human skin cells

Juewon Kim, Jeongin Lee, Hyung-June Kim, Hyunjung Choi

Table S1

Figure S1-4

Table S1. IPL settings and outputs

		Yellow		Green	
**Level**	**Lumens**	**Current (mA)**	**Duty (%)**	**Current (mA)**	**Duty (%)**
**1**	70	82	36	43	20
**2**	90	110	47	54	30
**3**	110	150	59	67	39
**4**	130	182	70	80	43
**5**	150	227	89	90	51

The settings of IPL device based on lumens. Actual output of IPL in current and duty was indicated under yellow and green settings.

A

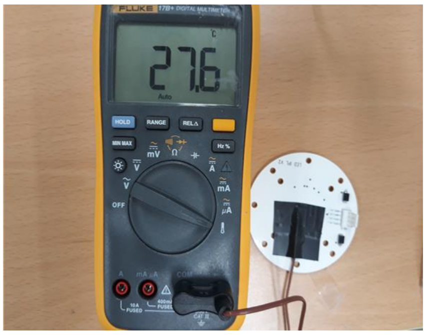

B

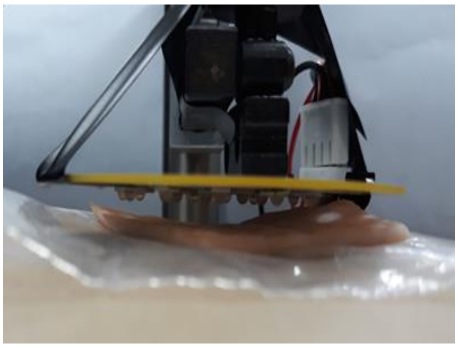

C

Time	0	1H 20min	2H	3H 20min	5H 21min
Temperature	26 ℃	26 ℃	26 ℃	26 ℃	26 ℃
Image	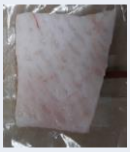	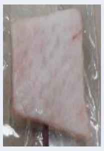	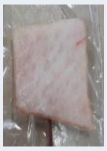	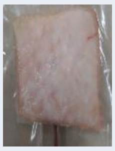	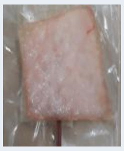

Figure S1. Temperature changes by IPL irradiation were estimated. (A) thermometer showing hand display unit (left) and sensor unit (right) with 60 mm^2 plate well setting. (B) temperature change degree of sequential irradiation was measured in IPL-treated swine skin model. (C) there was no significant change in temperature when IPL irradiated to swine skin for up to 5.21 hrs.

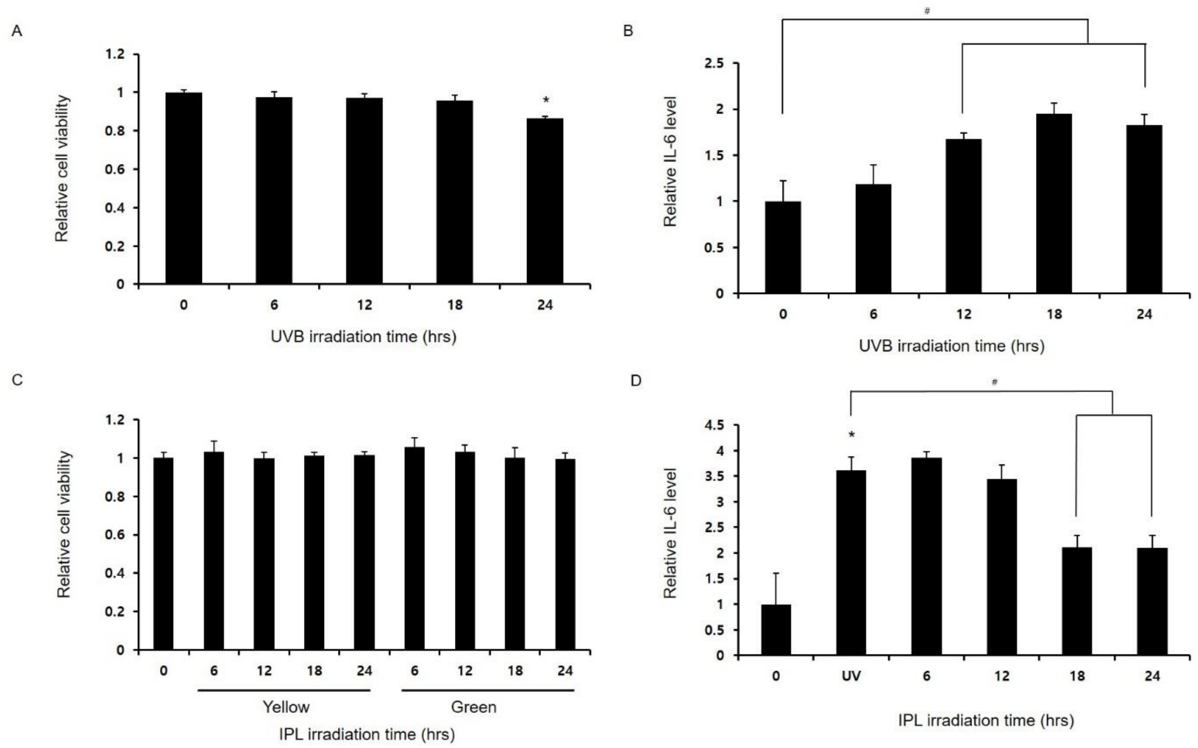

Fig. S2. Cell viability of NHEKs under treatment of UVB alone or IPL alone for 6 to 24 hrs. (A) Relative cell viability were not changed until 18 hrs irradiation of UVB (20 mJ/cm^2). (B) the secreted IL-6 level was checked for 24 hrs under UVB irradiation. (C) cell viability in yellow or green IPL application to the cells for 24 hrs. (D) relative levels of secreted IL-6 was examined at yellow IPL treatment for 24 hrs. The data are presented as the mean ± SD. (N = 3).

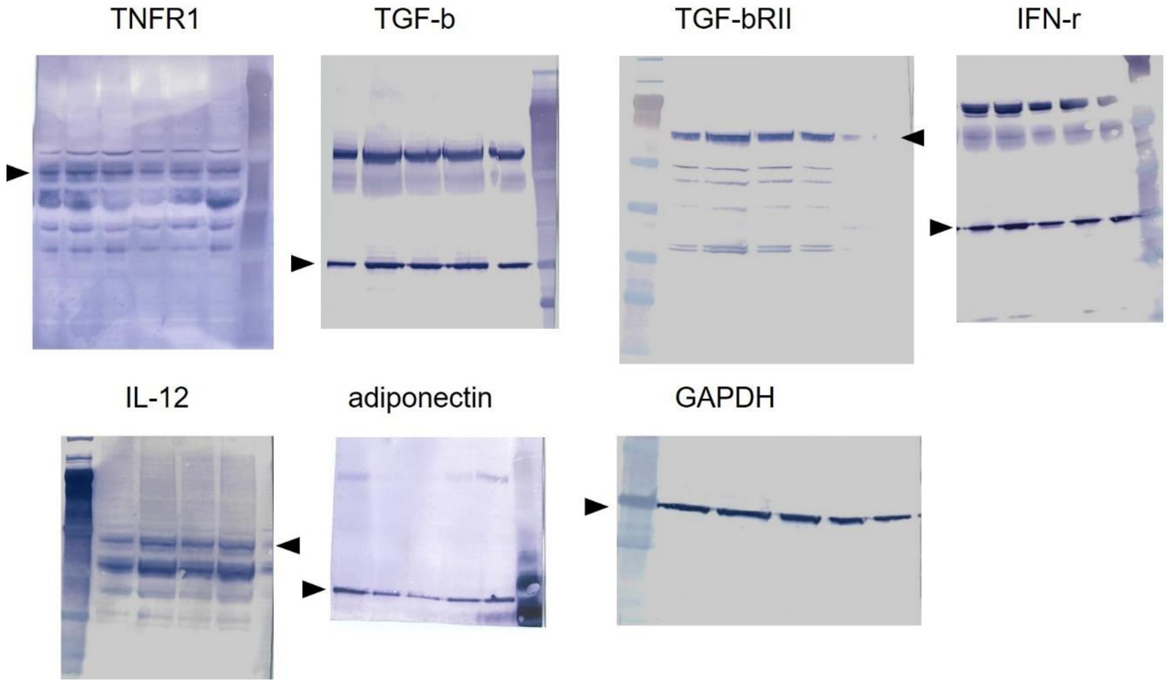

Figure S3. Full western blot image of Figure 3D.

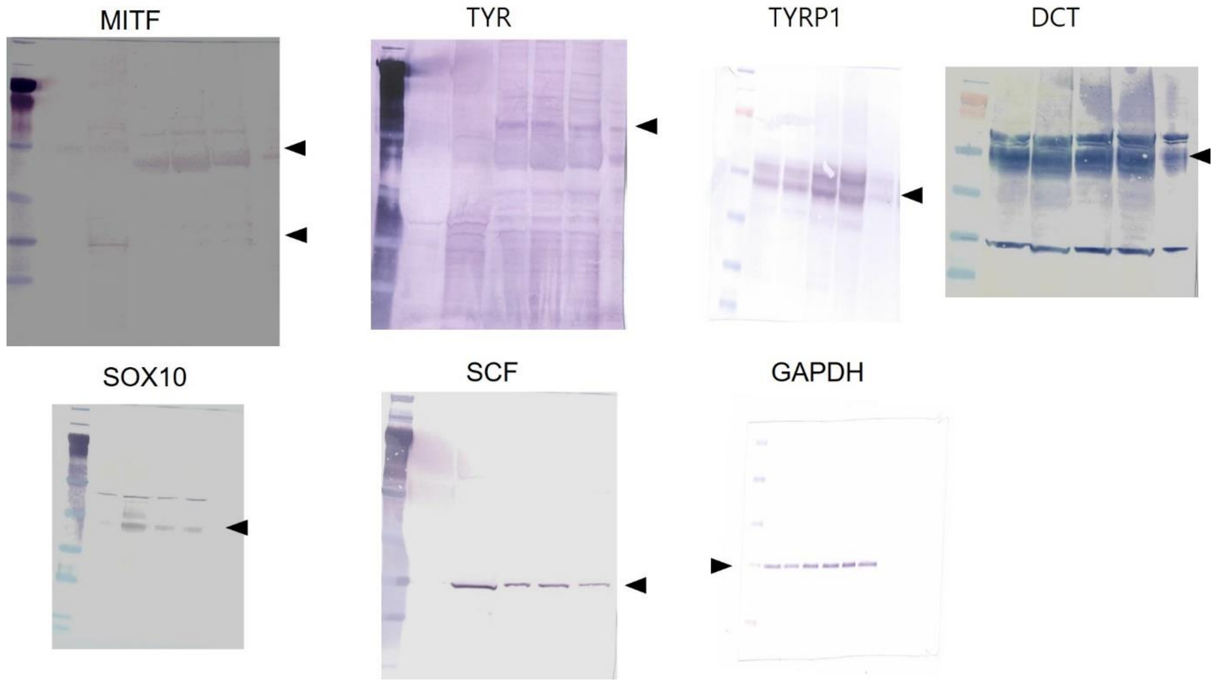

Figure S4. Full blot image of Figure 5B.
